# Leprosy—Presenting as Rheumatoid Arthritis Misleading the Correct Diagnosis

**DOI:** 10.1002/ccr3.70096

**Published:** 2025-01-10

**Authors:** Dhakal Sagar, Shrestha Kipa, Gurung Praful, Thapa Durga

**Affiliations:** ^1^ Department of Orthopaedics and Trauma Surgery Patan Academy of Health Sciences Lagankhel Lalitpur Nepal; ^2^ Amppipal Hospital Gorkha Nepal; ^3^ Kathmandu Medical College Sinamangal Kathmandu Nepal

**Keywords:** leprosy, MDT, mycobacterium, rheumatoid arthritis, ulnar clawing

## Abstract

Leprosy can present with symptoms resembling rheumatoid arthritis, leading to delays in diagnosis or inappropriate treatment, potentially progressing to lepromatous forms, neuropathy, and disability. Physicians must consider leprosy as a differential for rheumatoid arthritis, especially in endemic regions. Early detection is vital to prevent chronic neuropathy, disabilities, and disfigurement.

## 1 Introduction

Leprosy, also known as Hansen's disease, is a chronic granulomatous bacterial infection caused by *Mycobacterium leprae* bacilli, primarily affecting the skin and peripheral nerves. It can also impact the eyes, mucous membranes, bones, cartilage, joints, and testes, producing a spectrum of clinical presentations and the potential to cause severe disfigurement in affected individuals [1, 2, 3].

Elimination of leprosy as a public health problem was achieved globally in 2000, and in most countries, including Nepal, by 2010 [1, 4, 5, 6]. In 2020, a total of 127,396 new cases were reported worldwide, with a case detection rate of 16.4 per million population. Brazil, India, and Indonesia accounted for 72.5% of registered cases and 74% of new cases detected in 2020. In Nepal, 2304 new cases were detected in 2020 [4]. Diagnosis may be delayed due to unusual presentations or the disease mimicking other rheumatological disorders, especially when accompanied by polyarthritis [7, 8, 9, 10]. Early detection and treatment are critical for preventing neuropathy and disability.

Here we present the case of a 67‐year‐old male who experienced a delay in the diagnosis of leprosy, suffering for nearly 1.5 years due to its resemblance to seronegative rheumatoid arthritis and a lack of awareness regarding the variations in clinical presentation of leprosy.

## 2 Case History/Examination

A 67‐year‐old male from Gorkha, Nepal, visited the outpatient clinic at Amppipal Hospital, Gorkha with complaints of multiple joint pain for 1.5 years, decreased sensation in the left ring and little fingers for 8 months, and frequent ulcerated lesions on the left hypothenar, ring, and little fingers for 3 months.

For the past 1.5 years, he has experienced multiple joint pains, particularly in both hands (CMC, MCP, PIP > DIP) and elbows, worsening with rest associated with morning stiffness and occasional swelling. He gradually developed tingling, decreased sensation in his left ring and little fingers over the past 8 months, and reduced grip strength. He also had multiple turbid, fluid‐filled eruptions that later ulcerated on the hypothenar, ring, and little fingers. He has been taking antihypertensive medication (amlodipine 5 mg once a day) for 10 years and does not have diabetes or a history of traumatic injury to the elbow or cervical spine.

He visited the hospital multiple times and was evaluated for rheumatoid arthritis. Blood tests revealed a negative rheumatoid factor, leukocyte count of 6000/mm^3^ (23% lymphocytes, 65% neutrophils), hemoglobin of 12.8 g/dL, ESR of 18 mm/h, CRP negative, and uric acid level of 4.4 mg/dL. Suspecting seronegative rheumatoid arthritis, he was started on NSAIDs, methotrexate (15 mg once a week), and folic acid supplementation. For the ulcerated wounds, oral antibiotics such as cloxacillin 500 mg, flucloxacillin 500 mg four times a day and topical antibiotics like Ointment Mupirocin, Polysporin thrice a day were prescribed multiple times for 5–7 days from different centers likely with the intention to treat bacterial skin infections. The patient was non‐compliant with all medications due to recurring symptoms and further worsening of his condition.

**FIGURE 1:**
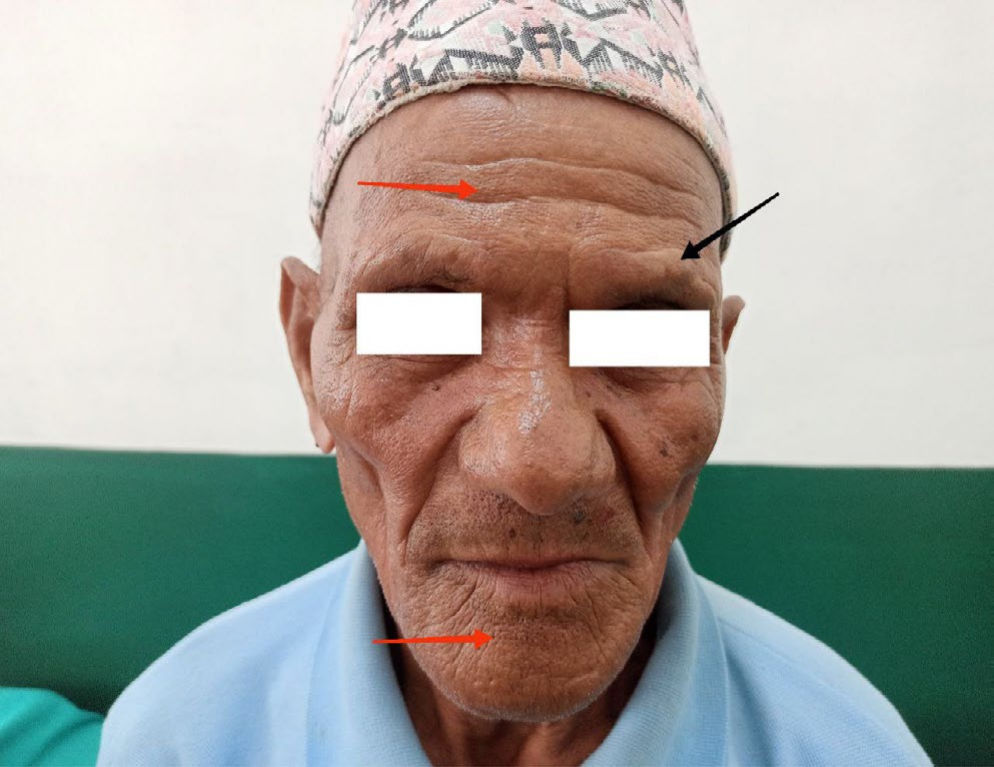
Leonine Facies, madarosis (black arrow), thickened cutaneous over forehead, nose and chin with prominent skin markings (red arrows).

**FIGURE 2:**
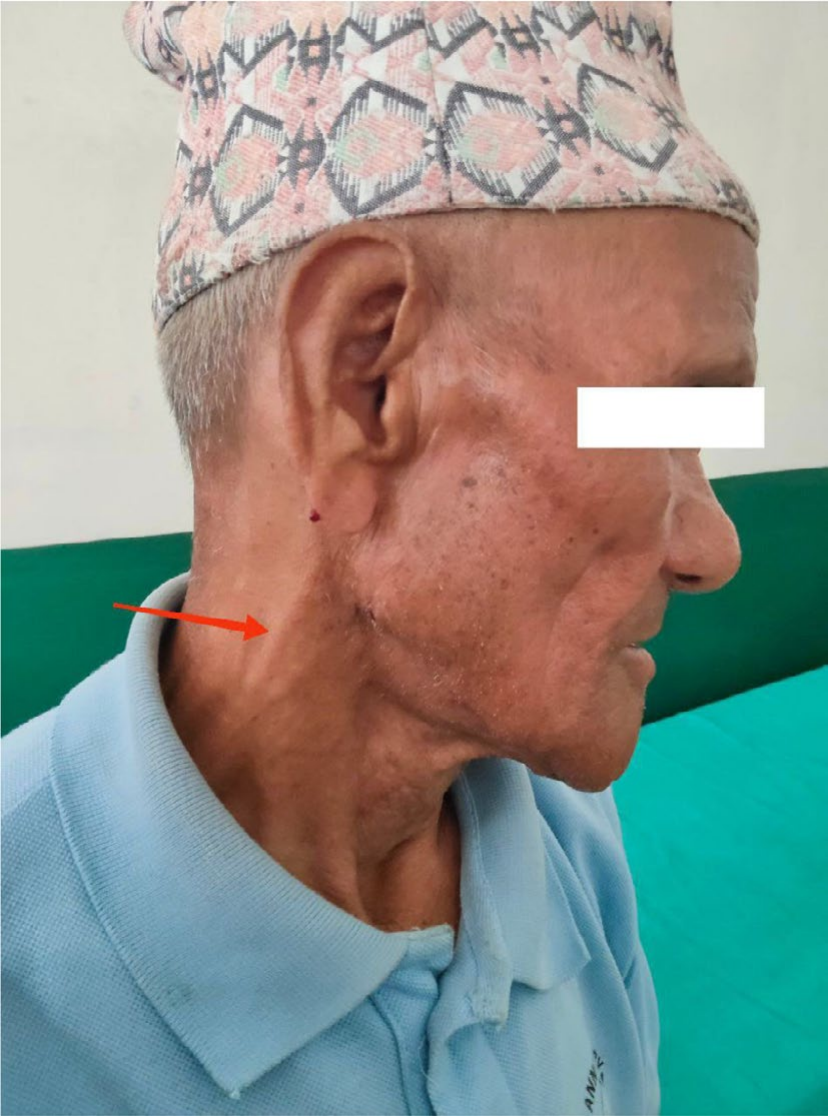
Prominent great auricular nerve (red arrow).

**FIGURE 3:**
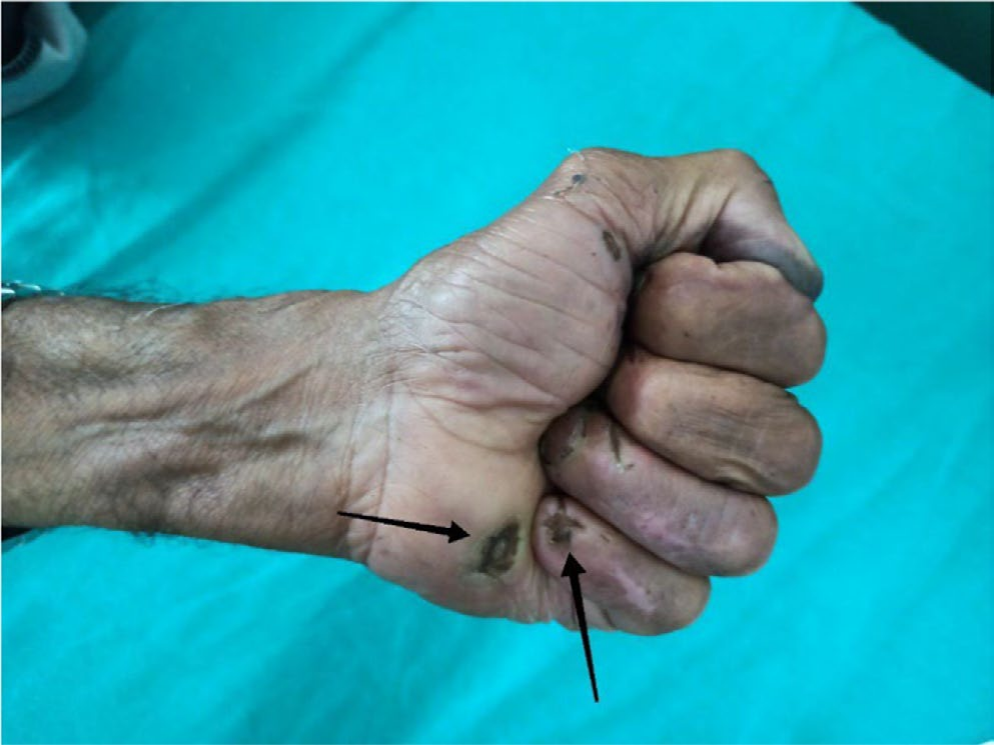
Healed ulcer over hypothenar, tip of fourth and fifth fingers (black arrow).

**FIGURE 4:**
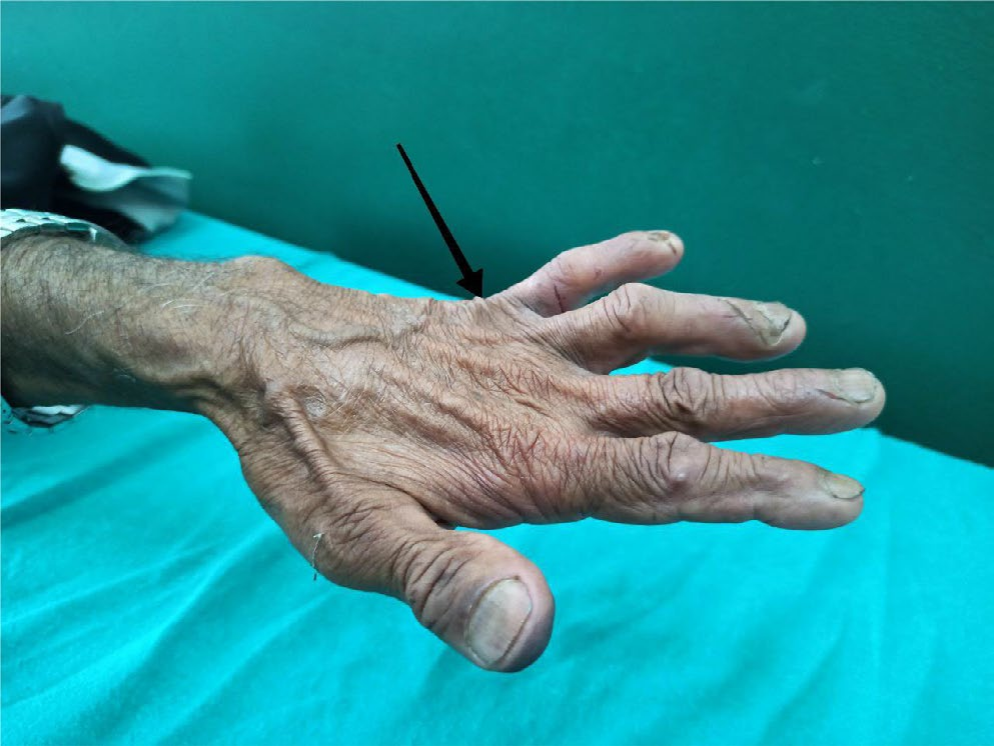
Ulnar clawing with interosseous atrophy (black arrow).

**FIGURE 5:**
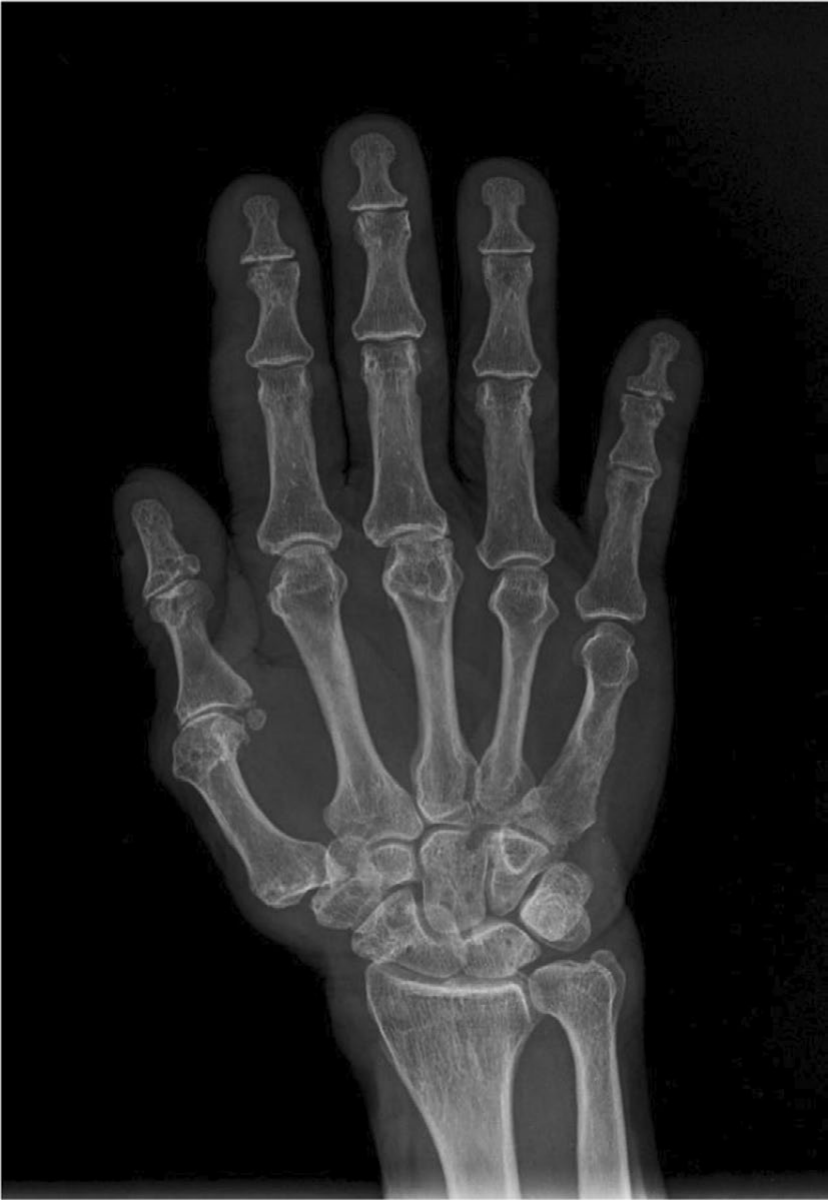
*AP radiograph of right hand with* juxta‐articular osteopenia with signs of erosion over the MCP, PIP, and DIP joints.

Upon re‐evaluation with a thorough history and examination, it was noted that he had decreased sensation over the forehead and facial area, along with a sensation of thick skin and changes in the shape of his nose. He had worked as a labourer in Bihar, India, for 20 years. His wife also experienced similar symptoms of multiple joint pain and ulcerated lesions over hands and was being treated for rheumatoid arthritis. However, there is no significant family history of leprosy. Ill‐defined erythematous plaques with minimal whitish scales were observed on the right retroauricular area, neck, left mid‐abdomen, left elbow, and forearm, with intact touch but impaired temperature sensitivity. Madarosis was noted, along with rough, thickened skin on the forehead, nose, and chin, resembling leonine facies (Figure 1). Enlarged bilateral greater auricular nerves with more prominence on the right than the left was observed (Figure 2), and prominent bilateral ulnar nerves, more on the left side were noticed. Atrophy of the interosseous, hypothenar, and thenar muscles was present, accompanied by ulnar clawing and ulcerated wounds at the tips of the little finger and hypothenar area (Figures 3 and 4). Signs of ulnar nerve injury, including the card test, froment's sign, and egawa test, were positive. A plain radiograph of the hand revealed juxta‐articular osteopenia with signs of erosion over the CMC, MCP, PIP, and DIP joints (Figure 5).

With these findings that was probably missed during his earlier hospital visits, leprosy was suspected. A slit skin smear was sent for diagnosis, which was positive with a bacteriological index of 1+. The diagnosis of lepromatous leprosy with left ulnar claw hand was made. Multidrug therapy (MDT) was initiated, consisting of a pulse dose of 600 mg rifampicin, 300 mg clofazimine, and 100 mg dapsone once a month, along with daily doses of 50 mg clofazimine and 100 mg dapsone. Proper physiotherapy was provided, and he received thorough counseling regarding drug reactions and potential complications.

## 3 Outcome and Follow‐Up

At the one‐month follow‐up, his skin lesions had completely disappeared, with no signs of drug reaction and a normal complete blood count, liver function tests, and renal function tests. Ulnar clawing and paraesthesia persisted, and he was advised to continue MDT and physiotherapy as instructed. For ulnar nerve palsy, an oral prednisolone 50 mg was started for a week, and tapering of dose by 10‐20 mg weekly was done. Further plan was made to continue MDT for a total of 12 months, administer low‐dose prednisolone (5–10 mg/day) for approximately 6 months for ulnar nerve lesion, and consider ulnar nerve decompression later if symptoms of ulnar nerve palsy persist in subsequent follow up.

## 4 Discussion

According to the World Health Organization (WHO), one of the following three features are essential for the diagnosis of leprosy:

- Definite loss of sensation in a pale (hypopigmented) or reddish skin patch.
- A thickened or enlarged peripheral nerve with loss of sensation.
- The presence of acid‐fast bacilli in a slit‐skin smear [5, 11].

Musculoskeletal symptoms, such as arthritis, are the third most common presenting manifestations of leprosy, following neurological and cutaneous features, and can mimic more common disorders like rheumatoid arthritis (RA) [7]. Clinicians should be aware of this disease, as some form of joint involvement is reported to occur in 75% of leprosy cases and may sometimes be the only obvious manifestation [9, 12]. Patients with leprosy have occasionally been treated for conditions such as systemic lupus erythematosus (SLE), RA, dermatopolymyositis, and systemic vasculitis [13]. Andrade et al. presented a case report of Brazilian man with polyarthritis and deformities, initially treated for rheumatoid arthritis for 5 years, was later diagnosed with lepromatous leprosy and received treatment for it [14]. Rath et al. presented a case of 35 year old lady of presented to a rheumatogical clinic with leprosy presenting with features similar to rheumatoid arthritis and so was initially treated as seronegative rheumatoid arthritis [15].

Slit‐skin smear positive is one of the essential criteria for the diagnosis, but it has low sensitivity of around 50%. Negative smear doesn't rule out the diagnosis. However bacterial index in slit skin smear helps to classify leprosy into WHO paucibacillary type with negative bacterial index and WHO multibacillary type with positive bacterial index [16, 17]. Biopsy with bacterial index of granuloma (BIG) was more sensitive and effective than slit skin smear test [17]. Multiplex PCR (M‐PCR), an alternative form of PCR specific to *M. leprae* DNA has been used for detection of smear negative clinical cases. M‐PCR reported a 70%–76% positive detection rate in indeterminate leprosy and even in the neuritic type of leprosy. The utility of M‐PCR for early diagnosis and household contact surveillance for leprosy is raising [18].

Leonine facies and madarosis are hallmarks of lepromatous leprosy [2]. Radiographic changes in RA are typically more pronounced than those in leprosy arthritis [19]. Early diagnosis and a full course of treatment are critical for preventing lifelong neuropathy and disability. Treatment consists of a combination of three drugs: dapsone, rifampicin, and clofazimine, administered for 6 months for paucibacillary and 12 months for multibacillary leprosy [5].

In patient with inflammatory rheumatic disorder prolonged use of steroid, immunosuppressive therapy, and biological agents may reactivate latent forms of tuberculosis and leprosy. Additionally, autoantibodies such as rheumatoid factor (RF) and antinuclear antibodies (ANA) may yield false positive result in leprosy [9]. However, when both RF and anti‐cyclic citrullinated peptide (anti‐CCP) are negative, other causes, including leprosy, should be considered especially if the patient is from an endemic area [12]. Therefore, not only can leprosy patients mimic rheumatoid disease, but patients with known rheumatoid disorders may also present with signs and symptoms of leprosy during treatment if they have a latent infection.

The ulnar nerve is the most commonly affected nerve in leprosy, with symptoms ranging from neuritis to clawing of the hand [20]. Steroid therapy, in conjunction with MDT, is the standard treatment for ulnar neuritis. Surgical decompression is required for patients who do not respond to 12 weeks of combination therapy with steroids or for those with severe ulnar neuritic pain that disrupts daily activities, particularly in cases involving nerve abscesses. The steroid dosage typically begins at 1 mg/kg/day, gradually tapering weekly, and is continued for 6–9 months, with a maintenance dose of 5–10 mg/day. In a study by Sajid et al., pain recovery was seen in 100% of cases, sensory recovery occurred in 60%–82% within 6–8 weeks, and motor improvement (reflected by upgrades in the Medical Research Council [MRC] score) was observed in 50%–80% of cases, depending on initial motor power, over a period of 24 to 54 weeks [20].

In our case, the patient initially presented with polyarthritis and was treated for seronegative rheumatoid arthritis. Due to persistent and worsening symptoms, he became noncompliant with his medication. After a few months, he began developing neuropathic symptoms in the ulnar distribution, followed by ulnar clawing and cutaneous lesions, which prompted suspicion of leprosy. Through a thorough history, examination, and slit skin smear, he was diagnosed with lepromatous leprosy, and treatment was initiated. The delay in diagnosis was due to misinterpreting the clinical signs as more indicative of rheumatoid arthritis and a lack of early suspicion of leprosy based on the timeline of symptoms. Therefore, it is essential to recognize the association of arthritis in leprosy, its resemblance to other inflammatory conditions, and the progression of symptoms for early detection and treatment.

## 5 Conclusion

Leprosy can often mimic rheumatoid arthritis, especially when patients present with polyarthralgia without additional evident signs or symptoms. Furthermore, individuals with other inflammatory conditions receiving DMARDs, biological agents, immunosuppressive therapy, or steroids may experience reactivation of latent infections, including tuberculosis and leprosy, particularly if they come from endemic regions. Therefore, physicians and rheumatologists should consider leprosy as a differential diagnosis when applicable. Early detection is crucial to prevent chronic neuropathy, physical and functional disabilities, and disfigurement in affected individuals.

## Author Contributions

**Dhakal Sagar:** conceptualization, data curation, formal analysis, investigation, methodology, project administration, resources, supervision, writing – original draft, writing – review and editing. **Shrestha Kipa:** conceptualization, resources, writing – review and editing. **Gurung Praful:** methodology, supervision, validation, writing – review and editing. **Thapa Durga:** conceptualization, supervision, writing – review and editing.

## Ethics Statement

Written informed consent was obtained from the patient for publication of this case report and any accompanying images. A copy of the written consent is available for review by the Editor‐in‐Chief of this journal.

## Conflicts of Interest

The authors declare no conflicts of interest.

## Data Availability

Data openly available in a public repository that issues datasets with DOIs.
